# Physiology, pathophysiology and (mal)adaptations to chronic apnoeic training: a state-of-the-art review

**DOI:** 10.1007/s00421-021-04664-x

**Published:** 2021-03-31

**Authors:** Antonis Elia, M. Gennser, P. S. Harlow, Matthew J. Lees

**Affiliations:** 1 Division of Environmental Physiology, School of Chemistry, Bioengineering and Health, KTH Royal Institute of Technology, Berzelius väg 13, Solna, SE-171 65 Stockholm, Sweden; 2 School of Life Sciences, University of Nottingham, University Park, Nottingham, UK; 3 Department of Physical Education and Sport Sciences, Faculty of Education and Health Sciences, University of Limerick, Limerick, Ireland

**Keywords:** Breath-hold diving, Apnoea, Spleen, Skeletal muscle, Bone health, Haematology

## Abstract

Breath-hold diving is an activity that humans have engaged in since antiquity to forage for resources, provide sustenance and to support military campaigns. In modern times, breath-hold diving continues to gain popularity and recognition as both a competitive and recreational sport. The continued progression of world records is somewhat remarkable, particularly given the extreme hypoxaemic and hypercapnic conditions, and hydrostatic pressures these athletes endure. However, there is abundant literature to suggest a large inter-individual variation in the apnoeic capabilities that is thus far not fully understood. In this review, we explore developments in apnoea physiology and delineate the traits and mechanisms that potentially underpin this variation. In addition, we sought to highlight the physiological (mal)adaptations associated with consistent breath-hold training. Breath-hold divers (BHDs) are evidenced to exhibit a more pronounced diving-response than non-divers, while elite BHDs (EBHDs) also display beneficial adaptations in both blood and skeletal muscle. Importantly, these physiological characteristics are documented to be primarily influenced by training-induced stimuli. BHDs are exposed to unique physiological and environmental stressors, and as such possess an ability to withstand acute cerebrovascular and neuronal strains. Whether these characteristics are also a result of training-induced adaptations or genetic predisposition is less certain. Although the long-term effects of regular breath-hold diving activity are yet to be holistically established, preliminary evidence has posed considerations for cognitive, neurological, renal and bone health in BHDs. These areas should be explored further in longitudinal studies to more confidently ascertain the long-term health implications of extreme breath-holding activity.

It seems, then, that there are divers also among the Trojans.

Homer, Iliad (ca 800 BC)

## Introduction

Breath-hold diving has been practiced for centuries, with reports dating back as far as ~ 800 BC (Davis 1934). Remarkably, in some parts of the world (i.e., North and South East Asia) breath-hold diving is still practiced as a means of harvesting food. These habitual breath-hold diving populations spend up to ~ 60% of their workday submerged underwater whilst performing repeated (up to ~ 140) short dives (less than ~ 60 s), at depths spanning 5–25 m (Hong et al. 1991, 1963; Hurford et al. 1990; Schagatay et al. 2011). While the number of dives per workday of these habitual breath-hold diving populations are impressive, the maximum depth and duration are less so when compared with modern competitive BHDs that repeatedly flirt with their absolute physiological limits (Table 1).

**Table 1:** Current world records as recognised by the AIDA (2021)

Discipline	World records	
	Men	Women
Static apnoea (minutes:seconds)	11:35	9:02
Dynamic apnoea with fins (meters)	300	257
Dynamic apnoea without fins (meters)	244	191
Constant weight with fins (meters)	130	107
Constant weight without fins (meters)	102	73
Free immersion (meters)	125	98
Variable weight (meters)	146	130
No limit (meters)	214	160

During maximal apnoeic attempts, competitive BHDs endure extreme hypoxaemic hypercapnia (i.e., arterial oxygen partial pressure [PaO_2_], 2.7 kPa [20 mmHg], arterial carbon dioxide partial pressure [PaCO_2_], 7.3 kPa [55 mmHg]) (Bain et al. 2016; Willie et al. 2015), whereas during depth diving they also encounter severe hydrostatic pressures and even greater PaCO_2_. However, a remarkably large variation in individual performance and physiological threshold exists. Recent advances in apnoea physiology may now provide a clearer understanding of these large inter-individual variations in apnoeic capabilities. Accordingly, the first aim of this review was to explore the physiological characteristics and underlying mechanisms that govern an individual’s apnoeic capabilities, and secondly, to delineate whether these inter-individual variations stem from training-induced stimuli and/or genetic predisposition.

Continued advances in world records bear testament to the exceptional capabilities of the human body (Table 1), yet the continued quest for greater performance raises safety concerns. Despite a plethora of research outlining the physiological responses that occur during and/or following maximal apnoeic attempts, there is a dearth of information regarding the possible health implications associated with exposure to apnoea-related activities. Considering the growing popularity of the sport and the increasing number of people pursuing breath-hold diving as a competitive and/or recreational activity, increased awareness regarding the possible maladaptation(s) of chronic apnoeic training is necessary from both safety and medical standpoints.

Therefore, the purpose of this review was to: (i) delineate the physiological characteristics and underlying mechanisms that govern an individual’s apnoeic capabilities, (ii) examine whether inter-individual variations in apnoeic capabilities stem from training-induced stimuli and/or genetic predisposition, and finally, (iii) assess the physiological and pathophysiological (mal)adaptations to chronic apnoeic training.

## Physiological attributes of breath-hold divers

The ability to suppress respiratory urges and attain long breath-hold durations is dependent on the collective contribution of (i) the capacity for oxygen storage, (ii) the efficacy of oxygen conservation and utilisation, and (iii) training experience, including an individual’s psychological tolerance toward the increasing breathing urge and continuously intensifying involuntary diaphragmatic movements. Accordingly, the following section will seek to explore the physiological traits/characteristics that govern an individual’s apnoeic capabilities and the underlying mechanisms that protect BHDs against hypoxaemic hypercapnia.

### Lung volume

In humans, the theoretical maximum breath-hold time, for apnoeas initiated after air breathing (21% oxygen), is determined by the body’s oxygen stores and the rate that these are consumed (Ferretti et al. 1991; Mithoefer 1959, 1965). Considering that during an apnoeic bout, aerobic metabolism is limited to the body’s finite oxygen stores (i.e., comprising of blood [~ 98% of oxygen bound to haemoglobin], skeletal muscle [myoglobin] and the lungs), a higher oxygen reservoir at the start of a maximal attempt will extend the aerobic dive limit, thus permitting longer apnoeas (Mithoefer 1959, 1965; Whitelaw et al. 1987). It is noteworthy that diving mammals possess extremely high oxygen stores in skeletal muscle and blood, both of which serve as strong predictors of their diving capabilities (Ponganis 2011), whereas in humans apnoeic time is greatly dependent on lung oxygen stores (Ferretti et al. 1991). Hence, factors that might contribute towards enhancing these qualities are certainly considered advantageous with respect to apnoeic performance.

**Fig. 1:**
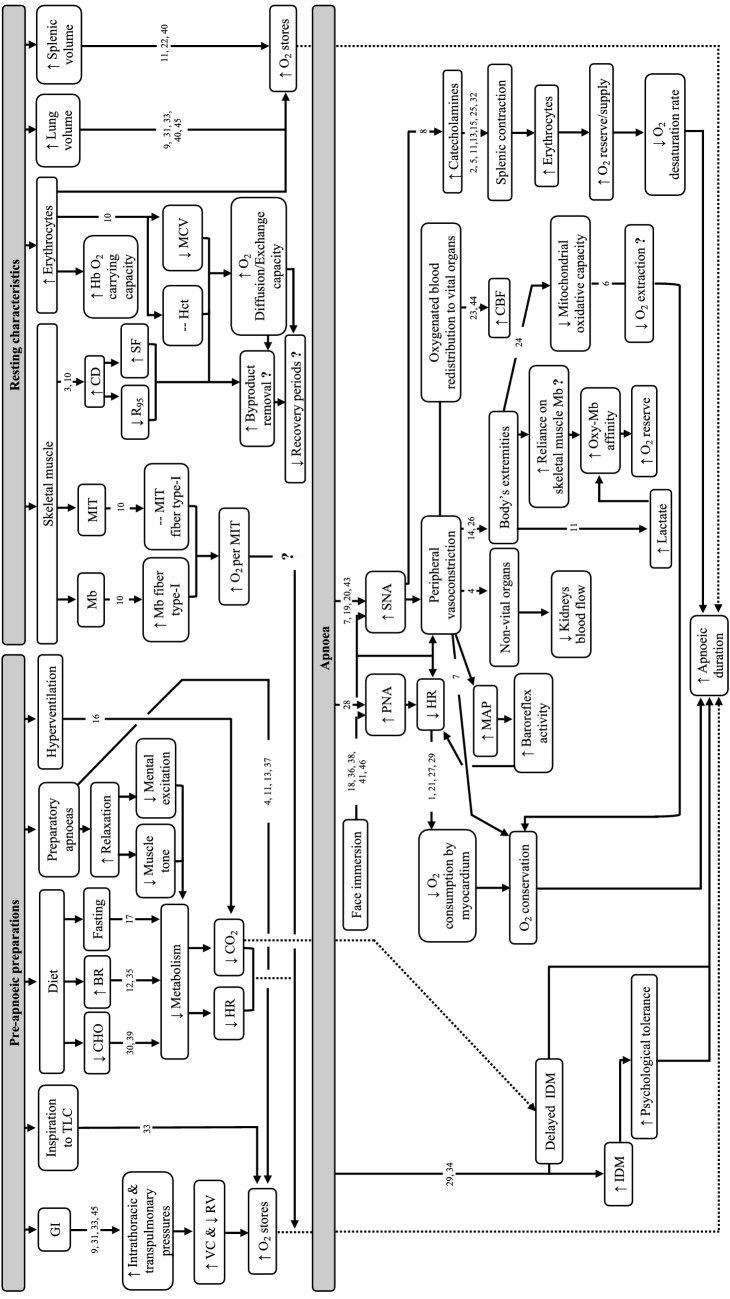
Schematic representation depicting: (i) pre-apnoeic strategies evidenced to contribute towards attaining longer apnoeic durations, (ii) the role of resting characteristics on apnoeic capabilities and (iii) the physiological modifications induced during static apnoeic attempts and their role in influencing apnoeic length. Arrows up (↑) and down (↓) within the framed box indicate an increase or decrease of the associated variable. Dotted arrow lines (▪▪▪▪▪▪) indicate the link between pre-apnoeic strategies/resting characteristics and apnoeic durations. Abbreviations: *BR* beetroot juice, *CBF* cerebral blood flow, *CD* capillary density, *CHO* carbohydrates, *CO*_*2*_  carbon dioxide, *GI* glossopharyngeal insufflation, *Hb* haemoglobin, *Hct* haematocrit, *HR* heart rate, *IDM* involuntary diaphragmatic movements, *MAP* mean arterial pressure, *Mb* myoglobin, *MCV* mean cell volume, *MIT* mitochondria, *O*_*2*_  oxygen, *PNA* parasympathetic nervous system, *R*_*95*_  diffusion distance, *RV* residual volume, *SF* sharing factor, *SNA* sympathetic nervous system, *TLC* total lung capacity, *VC* vital capacity. Supporting literature is denoted by the numbers, where; 1 = Asmussen and Kristiansson (1968), 2 = Ayers et al. (1972), 3 = Bae et al. (2003), 4 = Baković et al. (2003), 5 = Bakovic et al. (2013), 6 = Chicco et al. (2014), 7 = Dujic et al. (2008), 8 = Eichhorn et al. (2017), 9 = Eichhorn et al. (2018), 10 = Elia et al. (2019b), 11 = Elia et al. (2021b), 12 = Engan et al. (2012), 13 = Espersen et al. (2002), 14 = Ferretti (2001), 15 = Fredén et al. (1978), 16 = Gardner (1996), 17 = Ghiani et al. (2016), 18 = Hayashi et al. (1997), 19 = Heistad et al. (1968), 20 = Heusser et al. (2009), 21 = Hoiland et al. (2017), 22 = Ilardo et al. (2018), 23 = Joulia et al. (2009), 24 = Kjeld et al. (2018), 25 = Kutti et al. (1977), 26 = Kyhl et al. (2016), 27 = Landsberg (1975), 28 = Lemaitre et al. (2015), 29 = Lin (1982), 30 = Lindholm et al. (2007), 31 = Loring et al. (2007), 32 = Olsson et al. (1976), 33 = Overgaard et al. (2006), 34 = Palada et al. (2007), 35 = Patrician and Schagatay (2017), 36 = Paulev et al. (1990), 37 = Richardson et al. (2005), 38 = Schagatay and Holm (1996), 39 = Schagatay and Lodin-Sundström (2014), 40 = Schagatay et al. (2012), 41 = Shamsuzzaman et al. (2014), 42 = Steinback et al. (2010), 43 = Sterba and Lundgren (1988), 44 = Vestergaard and Larsson (2019), 45 = Whittaker and Irvin (2007)

Lung oxygen stores are governed by the combined contribution of inspired alveolar oxygen fraction and lung volume (Mithoefer 1965). For any given oxygen fraction in the alveoli, an individual’s lung volume is directly proportional to their oxygen stores and, concomitantly, to the aerobic energy resources that can be made available during an apnoeic attempt (Ferretti et al. 1991). It is, therefore, not surprising that competitive BHDs strive to commence their maximal apnoeic attempts at lung volumes in proximity to their total lung capacity (TLC). Thus far, several studies have documented the importance of lung volume on apnoeic duration (Andersson and Schagatay 1998; Mithoefer 1965; Muxworthy 1951; Overgaard et al. 2006; Whitelaw et al. 1987). To illustrate, apnoeas performed at TLC led to significantly longer apnoeic durations (309 ± 38 s) compared with apnoeas performed at 85% of vital capacity (297 ± 48 s) (Overgaard et al. 2006) (Fig. 1). These performance increments are ascribed to a higher oxygen reservoir being readily available to support aerobic metabolism, an attenuated oxygen desaturation rate, an increased carbon dioxide buffering capacity, as well as a delayed Hering–Breuer deflation reflex (Godfrey et al. 1969; Mithoefer 1959, 1965; Overgaard et al. 2006; Rose et al. 1979).

Presently, there is conflicting evidence regarding the lung volumes of competitive BHDs, with some studies reporting significantly greater volume in BHDs compared with non-divers (NDs) (Ferretti et al. 2012; Schaefer et al. 1968; Schagatay 2011; Stewart et al. 2005) and other work failing to report any differences (Andersson et al. 2009; Roecker et al. 2014; Tetzlaff et al. 2008). However, in most reports (including those quoted here), the TLC of elite BHDs (EBHD) exceeds the maximum range expected in healthy age-matched adults (i.e., male: 7.07 ± 1.60 L; female: 5.25 ± 0.76 L; Neder et al. 1999) (Table 2). These distinct respiratory characteristics may be ascribed to individual disposition, for example increased respiratory muscle strength and/or chest flexibility (Eichinger et al. 2008; Johansson and Schagatay 2012; Whittaker and Irvin 2007). Indeed, there is evidence suggesting that long-term (i.e., 6–11 weeks, 3–5 times per week) inspiratory muscle training, comprising glossopharyngeal insufflation and lung-stretching regimes, significantly enhances vital capacity by 0.13–0.45 L (Johansson and Schagatay 2012; Nygren-Bonnier et al. 2007). However, to what extent these increases stem from a reduction in pulmonary elastic recoil (i.e., due to reduced tension of elastic elements [e.g., elastin or collagen fibres] or from a change in surface forces from surfactant release form alveolar type-II cells) or chest wall recoil at high inspiratory volumes remains to be elucidated (Eichinger et al. 2008; Ferretti et al. 2012; Nygren-Bonnier et al. 2007; Rodarte et al. 1999; Seccombe et al. 2013, 2006; Tetzlaff et al. 2008; Whittaker and Irvin 2007). Notwithstanding, these training-induced adaptations may provide a partial explanation of the greater apnoeic capabilities witnessed in EBHDs compared with less trained BHDs and NDs, since a greater amount of oxygen will be readily available to support an apnoeic attempt.

**Table 2:** Lung volume and apnoeic performance characteristics of breath-hold divers

Reference	Participants	Apnoeic performance characteristics	TLC (L)
Schaefer et al. (1968)	1 EBHD (age: 33 years)	*PB Constant Weight Depth:* *66 m*	9.1
Muth et al. (2003)	2 EBHD	*PB STA:* 393 ± 41 s (364–422 s) *PB Constant Weight Depth:* 55 ± 7 m (50–60 m)	9 ± 1.41 (8–10)
Simpson et al. (2003)	1 EBHD	*PB STA:* ~ 480 s *PB DYN:* ~ 190 m *PB Constant Weight Depth:* ~ 90 m	9.28
Overgaard et al. (2006)	7 male BHD (age: 30 ± 2 years)	*Years practicing apnoea:* > 2 years	9.73 ± 1.36
Loring et al. (2007)	3 male and 1 female EBHD	*PB STA:* Male: 511 ± 85 s (405–608 s) Female: 376 s	Male: 9.04 ± 0.67 (8.88–9.78) Female: 5.91
Prommer et al. (2007)	7 male (age: 35 ± 9 years) and 3 female trained BHD (age: 32 ± 6 years)	*Years practicing apnoea:* > 3 years	Male: 10.58 ± 3.48 (6.4–12.8) Female: 8.8 ± 1.7 (7.6–10)
Walterspacher et al. (2011)	12 male EBHD	*PB STA:* 383 ± 47 s (304–469 s) *PB Depth:* 52 ± 29.4 m (32–125 m) *Years of competitive experience:* 6.6 ± 3.4 years (1–10 years)	9 ± 1.1 (7.36–10.82)
Ferretti et al. (2012)	8 male extreme BHD (age: 35 ± 4 years)	Assisted breath-hold diving of at least 50 m	8.76 ± 0.63 (7.3–10)
Stembridge et al. (2017)	14 male and 1 female EBHD	*PB STA:* 401 s (296–560 s) *Years of competitive experience:* 5.2 years (1.5–14 years)	8.28 ± 1.06

*BHD* breath-hold divers, *DYN* dynamic apnoea with fins, *EBHD* elite breath-hold divers, *ND* non-divers, *PB* personal best, *STA* static apnoea, *TLC* total lung capacity

#### Glossopharyngeal insufflation and apnoeic performance

The importance of lung oxygen stores in apnoeic performance is also instantiated by the use of glossopharyngeal insufflation.^1^ This manoeuvre acutely increases TLC by up to ~ 47% (Loring et al. 2007) prior to an apnoeic attempt, and markedly improves diving depth, dive duration, static and dynamic apnoeic performance (Lemaître et al. 2010; Lindholm and Nyrén 2005; Overgaard et al. 2006; Whitelaw et al. 1987). Glossopharyngeal insufflation at TLC induces increases in intrathoracic gas volume and transpulmonary pressure (i.e., up to 8 kPa [≈ 80 cmH_2_O]), with a maximum increase in intrapulmonary pressure of 108 cmH_2_O (Loring et al. 2007). The increased transpulmonary and intrapulmonary pressure lowers the amount of blood in the chest and provides more space for air (Whittaker and Irvin 2007; Eichinger et al. 2008). Approximately one-third of the additional air is accommodated by air compression and the remainder is facilitated by volume distension of the lungs (Seccombe et al. 2006). These observations on glossopharyngeal insufflation substantiate the impact that lung oxygen stores have on apnoeic performance. An individual’s ability to exceed their ‘normal’ TLC may also represent another factor that could potentially contribute towards the large inter-individual apnoeic performance variation reported across the literature.

### Splenic volume

The spleen is the largest lymphoid organ and is located beneath the 8th–11th thoracic rib on the left-hand side of the human body (Horan 2009). It is implicated in the process of erythrophagocytosis but also serves as an antibody production site and erythrocyte reservoir, with humans storing ~ 10% of their total erythrocyte volume in their spleens (Mebius and Kraal 2005; Stewart and McKenzie 2002). Evidence suggest that exercise interventions, hypercapnic and hypoxic/hypoxaemic conditions stimulate splenic contractions, with the latter serving as the most effective stimulus (Elia et al. 2021b; Laub et al. 1993; Otto et al. 2013; Richardson et al. 2012). Thus, following 3–5 repeated maximal apnoeic attempts the spleen contracts, releasing its stored erythrocytes into the systemic circulation (Elia et al. 2021b) (Fig. 1). These increases potentiate the oxygen binding and carrying capacity of blood; hence, the oxygen reserve is increased by the systemic mobilisation of erythrocytes. Thereby successive apnoeic bouts will commence with a greater amount of readily available oxygen, attenuating the oxygen desaturation rate and subsequently delaying the physiological breaking point, thus contributing towards an extended apnoeic duration (Bakovic et al. 2013; Schagatay et al. 2005) (Fig. 1). Hence, a larger splenic volume with the capacity to store a greater number of erythrocytes is considered advantageous for apnoeic performance (Elia et al. 2021b; Schagatay et al. 2012) (Fig. 1). To date, no cross-sectional study has found any splenic volume differences between diving and non-diving populations (Baković et al. 2003; Elia et al. 2019b, 2021b; Hurford et al. 1990; Prommer et al. 2007) (Table 3). Interestingly, both Schagatay et al. (2012) and Elia et al. (2019b) observed that in the most successful BHDs, splenic volume appears to exceed the normal range (i.e., 455–598 mL and 402–499 mL, respectively) documented in healthy adult males (76–400 mL; mean = 238 ± 70 mL) (Geraghty et al. 2004). Whether this characteristic is coherently shared across the most successful BHDs remains to be determined.

**Table 3:** Splenic volumes (quantified by means of ultrasonography) and performance characteristics of breath-hold divers

References	Participants	Performance characteristics	Formula	Splenic volume (mL)
Hurford et al. (1990)	10 female Korean Ama habitual BHD (age: 38–60 years) and 3 female control (age: 24 ± 1 years)	*Years practicing apnoea:* 34 ± 7 years Length of diving shifts 174 ± 46 min	Cross-sectional area = 0.8 × (length × width) Volume = (7.53 × cross-sectional area) − 77.56	BHD: 206 ± 49 Control: 223 ± 48
Baković et al. (2003)	10 male trained BHD (age: 28.6 ± 1.7 years) and 10 male untrained individuals (age: 27.8 ± 2.4 years)	–		BHD: 344.1 ± 16.6 ND: 332 ± 25.1
Prommer et al. (2007)	7 male (age: 35 ± 9 years) and 3 female trained BHD (age: 32 ± 6 years), and 7 male Scuba divers (age: 38 ± 11 years)	*Years practicing apnoea:* > 3 years *Training background:* 2—3 h, 3 times per week or more		BHD: 191 ± 47 Scuba divers: 229 ± 55
Palada et al. (2007)	7 experienced BHD (age: 27.4 ± 4.6 years)	*Years practicing apnoea:* 7.1 ± 3.6 years (5–14) *PB STA:* 284.0 ± 34.4 s (240–335) *PB Constant Weight Depth*: 33.7 ± 5.6 m (25–40 m)		308 ± 135
Palada et al. (2008)	7 male BHD (age: 27 ± 5 years)	*Years practicing apnoea:* 7 ± 4 years (5–14 years) *PB STA:* 284 ± 34 s (240–335 s) *PB Constant Weight Depth:* 34 ± 6 m (25–40 m)		283 ± 76
Schagatay et al. (2012)	14 male EBHD (age: 29 ± 2 years)	*Years practicing apnoea:* 5.8 ± 1.2 years *Training background:* 6.2 ± 0.6 h per week	Pilström formula Volume = (*Lπ*[*WT*−*T*^2^]/3)	336 ± 32 (215–598)
Elia et al. (2019b)	11 male EBHD and 10 male matched control ND	*Years practicing apnoea:* 7 ± 2 years *Training background:* 8 ± 2 h per week *PB STA:* 414 ± 101 s *PB DYN:* 202 ± 46 m *PB DNF:* 145 ± 50 m		BHD: 300 ± 122 (128–499) ND: 297 ± 77 (198–452)

*BHD* breath-hold divers, *DNF* dynamic apnoea no fins, *DYN* dynamic apnoea with fins, *EBHD* elite breath-hold divers, *L* length, *ND* non-divers, *PB* personal best, *STA* static apnoea, *T* thickness, *W* width

Reports on associations between splenic volume and bodyweight, height, gender and/or age are mixed (Elia et al. 2019b; Prassopoulos et al. 1997; Schagatay et al. 2012; Spielmann et al. 2005), suggesting that there may be greater individual variation than in other organs. A comparative genomic study by Ilardo et al. (2018) demonstrated that splenic volume is not governed by a training-induced stimulus. Rather, it is partly governed by a natural selection on genetic variants in the PDE10A gene. Contrastingly, recent work by Bouten et al. (2019) recorded significant increases in splenic volume (+ 24%) following 8-weeks of static apnoeic training (i.e., five apnoeic bouts per day). Although the underpinning mechanisms dictating splenic volume are presently unclear, collectively these studies indicate that splenic size may be governed by a complex interplay between hypoxic/hypoxaemic training and genetics. Further research is necessary to fully elucidate the mechanisms underpinning splenic size and growth.

### Haematological indices

The lack of respiratory exchange during voluntary apnoea necessitates a reliance upon the body’s finite oxygen resources. In marine mammals, a high haemoglobin concentration is considered a beneficial adaptation to apnoeic diving, with a number of studies denoting a direct relationship between haemoglobin concentrations and diving capabilities (Ponganis 2011). Contrarily, this relationship between haemoglobin and apnoeic capacities does not seem to be shared by humans. To date, numerous studies have examined the resting haematological characteristics of habitual and competitive BHDs, with some reporting higher red blood cell (RBC) counts and/or haemoglobin concentrations in divers, while others have failed to record any differences compared with non-diving populations (Table 4). Although these discrepancies may partly be related to the training status of the BHDs recruited, it is worth mentioning that both the RBC and haemoglobin concentrations observed in BHDs lie within the physiological range expected in healthy adults (Osei-Bimpong et al. 2012).

**Table 4:** Resting haematological characteristics of breath-hold divers

References	Participants	Performance characteristics	Red blood cells	Haemoglobin	Haematocrit	Reticulocytes
Kang et al. (1963)	20 female habitual BHD (Ama) and 20 female ND	–	BHD:3.53 ± 0.05 × 10^6^/mm^3^ ND: 3.63 ± 0.12 × 10^6^/mm^3^	BHD: 12.8 ± 0.2 g/100 ml* ND: 11.9 ± 0.3 g/100 ml	BHD: 35.1 ± 0.6% ND: 34.6 ± 0.7%	–
Ferretti et al. (1991)	1 male (age: 57 years) and 2 female EBHD (mean age: 29 years)	*PB Depth No limits category:* Male 101 m, and female 70 m and 80 m	–	Male: 148 g/L Female: 143 g/L and 137 g/L	–	–
Qvist et al. (1993)	5 female Korean Ama (age: 38–52 years)	Perform repeated dives to depths of 3–7 m for up to 4 h/day, spending 25–30% of this time underwater and completing > 100 dives during a single workday	–	12.8 ± 0.3 g/dl	38.5 ± 0.6%	–
Baković et al. (2003)	10 male trained BHD (age: 28.6 ± 1.7 years) and 10 male untrained individuals (age: 27.8 ± 2.4 years)	Four were national team members	–	–	BHD: 41.3 ± 0.01% Untrained: 43.8 ± 0.01%	–
Richardson et al. (2005)	29 male and 4 female EBHD (age: 30.3 ± 10.6 years), 23 elite male skiers (age: 20.2 ± 3 years), 23 male and 9 female untrained individuals (age: 30 ± 6.3 years)	*Training background:* 5.5 ± 4 h per week	–	EBHD: 150.1 g/L Skiers: 145.5 g/L Untrained: 146.9 g/L	–	–
Bakovic et al. (2005)	18 trained male BHD and 18 male ND	*Years practicing apnoea:* 9.5 years (4–26 years)	–	BHD: 147.7 (125–177 g/L) ND: 147.9 (131–172 g/L)	BHD: 43% (36–53%) ND: 43% (39–49%)	–
Stewart et al. (2005)	10 trained BHD (age: 36 ± 11.3 years) and 10 ND (age: 32 ± 9.5 years)	*Years practicing apnoea:* 17 years	–	BHD: 14.4 ± 1.6 g/dl ND: 14.2 ± 0.9 g/dl	BHD: 44.3 ± 5.6% ND: 40.6 ± 4.4%	–
Prommer et al. (2007)	7 male (age: 35 ± 9 years) and 3 female trained BHD (age: 32 ± 6 years), 7 male Scuba divers (age: 38 ± 11 years), 10 male untrained ND (age: 31 ± 10 years) and 9 non-diving triathletes (age: 31 ± 8 years)	*Training background:* 2–3 h, 3 times per week or more over at least the 3 years	–	BHD male: 152 ± 5 g/L BHD female: 141 ± 9 g/L Scuba divers: 154 ± 9 g/L Untrained ND: 151 ± 8 g/L Triathletes: 152 ± 10 g/L	–	–
Schagatay et al. (2007)	7 male BHD (age mean: 27 years [22–35 years])	–	–	14.18 ± 0.48 g/dl	43.5 ± 1.3%	–
Kjeld et al. (2009)	8 male and 1 female trained competitive BHD, 5 male and 1 ND	Current or former national record holders	–	142 g/L	–	–
Richardson et al. (2009)	10 male BHD	*Training background:* < 2 h per week	–	134.1 ± 0.8 g/L	40.9 ± 2%	–
Sureda et al. (2015)	7 male professional spearfishing BHD (age: 30 ± 2.1 years)	*Years practicing apnoea:* 5—20 years *Training background:* 3–4 h diving 5 times per week	4.93 ± 0.07 × 10^12^/L	14.7 ± 0.1 g/dl	42.8 ± 0.4%	–
Oh et al. (2017)	715 female Haenyeo habitual BHD and 715 matched ND	–	–	BHD: 13.0 ± 1.2 g/dl ND: 13.0 ± 1.1 g/dl	–	–
Fernandez et al. (2017)	56 male BHD (age: 36.6 ± 8.5 years [22–57 years])	*Years practicing apnoea*: 2.09 ± 0.7 years	–	15.6 ± 1.1 g/dl (14.1—17.7 g/dl)	–	–
Kjeld et al. (2018)	8 male BHD (age: 42 ± 8 years) and 8 male judo athletes (age: 36 ± 11years)	*PB STA:* 403 ± 62 s *PB DYN:* 163 ± 36 m *PB DNF:* 133 ± 37 m	–	BHD: 15.2 ± 0.8 g/dl ND: 15.3 ± 0.8 g/dl	–	–
Puspitaningrum et al. (2019)	21 male habitual BHD (Bajau) (age: 15–62 years)	–	–	15.62 g/dl (12.9–16.9 g/dl)	–	–
Elia et al. (2019b)	11 male EBHD and 10 male matched control ND	*Years practicing apnoea:* 7 ± 2 years *Training background:* 8 ± 2 h per week *PB STA:* 414 ± 101 s *PB DYN:* 202 ± 46 m *PB DNF:* 145 ± 50 m	EBHD: 5.43 ± 0.49 •10^12^/L* (4.80–6.3 × 10^12^/L) ND: 4.93 ± 0.24 × 10^12^/L (4.64–5.34 × 10^12^/L)	EBHD: 153 ± 6 g/L* (144–161 g/L) ND: 148 ± 3 g/L (145–152 g/L)	EBHD: 46 ± 4% (42–52%) ND: 45 ± 2% (43–49%)	EBHD: 1.47 ± 0.29%* (1.1–2%) ND: 1.13 ± 0.30% (0.7–1.6%)
Marlinge et al. (2019)	12 male experienced BHD (age: 37 ± 8 years), 12 male experienced Scuba divers (age: 51 ± 10 years) and 8 male ND (age: 48 ± 4 years)	–	–	BHD: 15 ± 0.3 g/dl Scuba: 15.2 ± 1.5 g/dl Control: 15.3 ± 0.9 g/dl	BHD: 45 ± 0.9% Scuba: 44.9 ± 1.2% Control: 45.5 ± 1.8%	–
Fernandez et al. (2019)	29 male BHD (age: 36 ± 5 years)	*Years practicing apnoea:* mean of 2 years	–	15.9 ± 1.2 g/dl 15.4 ± 1.2 g/dl 15.5 ± 0.8 g/dl	–	–
Elia et al. (2021b)	8 male EBHD and 10 male ND	*Years practicing apnoea:* 6 ± 2 years *Training background:* 9 ± 1 h per week *PB STA:* 373 ± 35 s *PB DYN:* 133 ± 42 m	–	EBHD: 148 ± 6 g/L ND: 149 ± 4 g/L	EBHD: 45 ± 2% ND: 45 ± 2%	–

* BHD* breath-hold divers, *DNF* dynamic apnoea no fins, *DYN* dynamic apnoea with fins, *EBHD* elite breath-hold divers, *ND* non-divers, *PB* personal best, *STA* static apnoea

*denotes significance (*p*<0.05) between groups

Recently, Elia et al. (2019b) observed lower mean cell volume (MCV) in EBHD compared with ND (86 ± 6 fL vs*.* 92 ± 4 fL), findings that were situated at the lower range observed in healthy adult males of a similar age (83–101 fL) (Osei-Bimpong et al. 2012) (Table 4). Interestingly, a lower MCV has also been reported in both Chilean and Nepalese high-altitude natives (3700 m) (85 fL and 82.9 fL, respectively) (Winslow et al. 1989). This observation may suggest that the total surface area of RBCs is larger in EBHD (Holland and Forster 1966) (Fig. 1). A larger RBC volume increases the intracellular diffusion path, with a concomitant decrease in the permeability of the RBC membrane (Holland and Forster 1966). Additionally, Vandegriff and Olson (1984) demonstrated that oxygen release was less dependent on RBC size and shape than oxygen uptake. Thus, the lower MCV reported in the EBHD group might be advantageous, as haemoglobin will have a greater surface area to bind oxygen. However, whether this is a result of training-induced adaptations and/or is consistent across EBHD remains to be determined.

### Skeletal muscle

#### Capillarisation and fibre type characteristics

At present, only a small number of studies have explored the skeletal muscle characteristics of BHDs using established wet-lab techniques. Work by Bae et al. (2003) in Korean female BHDs documented higher capillary density (~ 39%) and smaller fibre cross-sectional area (CSA) in type-I (~ 22%), type-IIa (~ 33%) and type-IIx (~ 25%) muscle fibres compared to active controls, as well as a smaller CSA of type-II fibres compared with type-I fibres in BHDs. The percentage distribution of type-I fibres was similar between groups (BHDs vs*.* NDs), with type-II fibres the predominant fibre type. The proportion of type-IIx fibres was higher in BHDs (31%) compared with controls (22%). Subsequent work by Park et al. (2005) in Indonesian habitual BHDs documented similar muscle fibre type composition (i.e., in type-I and type-IIa, but not in type-IIx) and CSA to that of non-diving age-matched controls. A possible explanation to the morphological disparities (i.e., fibre type distribution and CSA) across these habitual diving populations may be the water temperatures (i.e., Korean BHDs, 10–27 °C; Indonesian BHDs, 29–30 °C) they are continuously exposed to during their daily working routines (Bae et al. 2003; Park et al. 2005). In rats, cold water immersion to ~ 20 °C for 1 h per day, 5 days per week, for 19-weeks significantly increased the number of type-II fibres (+ 158%) and reduced the number of type-I fibres (− 24%) (Walters and ConsTable 1993). Similarly, in cold-acclimatised rats that had been reared continuously for 68 generations in a 5 °C environment, a significantly smaller CSA of the soleus muscles was observed compared with unacclimated controls (Suzuki et al. 1997). Therefore, the greater proportion of type-II fibres alongside the smaller fibre CSA documented in the Korean BHD population may be a chronic adaptive response to cold-water immersion. However, the question then arises as to what is the benefit of such morphological adaptations? In carp, Rome et al. (1984) demonstrated that more muscle fibres, a greater proportion of which being type-II fibres, are recruited at lower temperatures (i.e., 10 °C compared with 20 °C) to compensate for the influence of temperature on muscle function and also to generate the power necessary for swimming. Therefore, one might speculate that the skeletal muscle characteristics of the Korean habitual BHDs may be beneficial in sustaining the efficacy of heat production in muscle tissues during exposure to cold environments; however, further research is necessary to affirm or refute this hypothesis.

A cross-sectional study by Elia et al. (2019b) recorded similar fibre type distribution between EBHDs and NDs (type-I: 56 ± 12% vs. 55 ± 13%; type-II: 44 ± 12% vs. 45 ± 13%), findings that aligned with Kjeld et al. (2018). Furthermore, the CSA across fibre types was comparable to the findings of Park et al. (2005) findings. Additionally, a higher capillary density, capillary-to-fibre-ratio, and a lower diffusion distance (*R*_95_) and sharing factor was reported in the skeletal muscle of EBHDs (Elia et al. 2019b). These findings are indicative of an enhanced blood to skeletal muscle fibre exchange capacity (Richardson et al. 1994; Saltin 1985) (Fig. 1). Thus, *ceteris paribus*, the rate at which oxygen enters the muscle fibre and by-products are removed during exercise is accelerated (McGuire and Secomb 2003; Tesch et al. 1981; Tesch and Wright 1983). Hence, in an apnoeic context, these capillarisation characteristics may confer advantages during recovery periods.

The capillarisation data of Elia et al. (2019b) (i.e., lower R_95_ and higher capillary density) concur with those previously reported in female Korean BHDs (Bae et al. 2003). The greater capillarisation observed in EBHDs might be attributable to their habitual apnoeic training, whereby periods of static and dynamic apnoeas are frequently repeated. It is well accepted that hypoxia serves a vital role in the regulation and expression of vascular endothelial growth factor (Breen et al. 1996; Gustafsson and Sundberg 2000), and consequently, the initiation of capillary neo-formation and angiogenesis (Arany et al. 2008; Desplanches et al. 1993; Kon et al. 2014; Terrados et al. 1990). Therefore, the greater capillarisation observed in BHDs may originate from the interaction between hypoxia and muscle recruitment occurring during apnoeic training.

#### Myoglobin

A high myoglobin concentration is regarded as an important adaptation to apnoeic diving in mammals and is closely related to apnoeic and diving capabilities (see review by Ponganis 2011). Myoglobin facilitates oxygen delivery to the mitochondria during periods of increased metabolic activity, and serves as an oxygen reservoir during times when ventilation ceases and hypoxia ensues (Postnikova and Shekhovtsova 2013). For instance, when skeletal muscle activity increases and intramyocellular oxygen levels begin to decline as a result of increased contractile activity (e.g., during dynamic apnoea), myoglobin supports intramyocellular oxygen by releasing its own bound oxygen, thus making it available for aerobic metabolism (Hoppeler and Vogt 2001; Kanatous et al. 2002, 2008, 2009). As a result, the active skeletal muscle can rely mainly on stored oxygen to sustain aerobic metabolism, permitting extended dive durations (Kooyman and Ponganis 1998; Polasek and Davis 2001). Thus, myoglobin serves a central role in balancing intracellular hypoxia and aerobic metabolism in response to exercise (Postnikova and Shekhovtsova 2013).

**Fig. 2:**
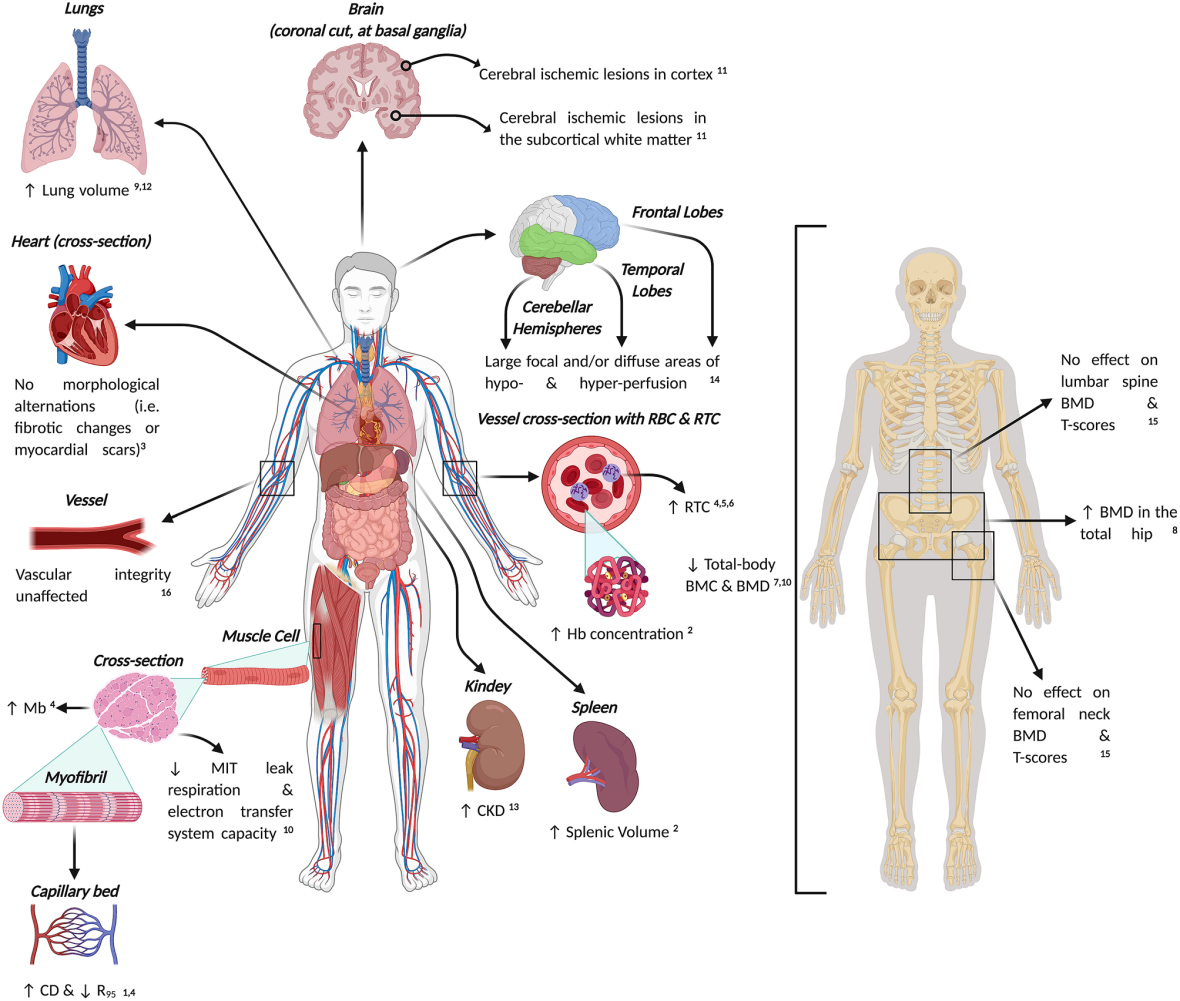
Schematic representation depicting current knowledge concerning the (mal)adaptations associated with long-term exposures to apnoea-related activities. Abbreviations: *BMC,* bone mineral content; *BMD,* bone mineral density; *CD,* capillary density; *CKD,* chronic kidney disease; *Hb,* haemoglobin; *Mb,* myoglobin; *MIT,* mitochondria; *RBC,* red blood cell; *RTC,* reticulocyte; *R*_*95*_ , diffusion distance. Supporting literature is denoted by the numbers, where; 1 = Bae et al. (2003), 2 = Bouten et al. (2019), 3 = Doerner et al. (2018), 4 = Elia et al. (2019b), 5 = Elia et al. (2020), 6 = Engan et al. (2013), 7 = Fernandez et al. (2017), 8 = Hwang et al. (2006), 9 = Johansson and Schagatay (2012), 10 = Kjeld et al. (2018), 11 = Kohshi et al. (2014), 12 = Nygren-Bonnier et al. (2007), 13 = Oh et al. (2017), 14 = Potkin and Uzsler (2006), 15 = Seo et al. (2018), 16 = Tanaka et al. (2016).  Figure created with BioRender.com

More recently, using an immunofluorescence microscopy technique, Elia et al. (2019b) found a greater type-I fibre myoglobin content in EBHDs (+ 27%) compared to NDs, but no difference in type-II fibre myoglobin content (Figs. 1, 2). The greater myoglobin stores reported in the EBHD group might be an important adaptation to apnoeic diving, as a greater skeletal muscle oxygen reserve will be readily available to support aerobic metabolism during apnoeic activity. These characteristics likely stem from an apnoea-specific, training-induced stimuli (Terrados et al. 1990) rather than genetic polymorphisms (Moore et al. 2002). Indeed, hypoxia coupled with skeletal muscle activation in both humans (Terrados et al. 1990), diving mammals and rodents has been documented to enhance myoglobin concentration in a muscle-specific manner (Dolar et al. 1999; Kanatous et al. 2008; Kanatous and Mammen 2010; Ponganis et al. 2010). Conversely, both hypoxic exposure and skeletal muscle activation in normoxic conditions have been shown to impair these adaptive myoglobin responses (Jacobs et al. 1987; Masuda et al. 1999; Terrados et al. 1990). Therefore, the higher myoglobin concentrations recorded in the EBHD group may stem from training-induced stimuli and could potentially, in a similar manner to diving mammals, provide an advantage during apnoeic activities.

#### Mitochondrial content

Our knowledge of mitochondria has evolved over the previous century, moving beyond understanding of their function in energy production to revealing their crucial roles as redox and apoptotic signal transducers within the cell (Kamga et al. 2012). In pinnipeds, average mitochondrial volume density values were 1.7- to 2.0-fold greater in the swimming muscles compared with the non-swimming muscles (Kanatous et al. 1999). This adaptation, allied to the lower aerobic capacity when compared to other long-duration divers (i.e., Weddell seals), suggests an ability to maintain low levels of aerobic lipid-based metabolism and reflects their energy-conserving modes of locomotion in a hypoxaemic environment (Kanatous et al. 2002, 1999).

In humans, a comparison of EBHDs and NDs found that mitochondrial complex subunits did not differ, nor did maximal citrate synthase or 3-hydroxyacyl CoA dehydrogenase activity (Kjeld et al. 2018) (Fig. 2). There were also no differences in markers of glucose metabolism or cytosolic antioxidant capacity. However, BHDs demonstrated lower mitochondrial leak respiration and electron transfer system capacity, and higher H_2_O_2_ emission during leak respiration than controls. These findings suggest that, similar to previous observations in diving mammals (Chicco et al. 2014), EBHDs possess lower mitochondrial oxidative capacity than NDs (Kjeld et al. 2018), which might represent an oxygen-sparing mechanism given the reduced skeletal muscle oxygen extraction during diving activity (Chicco et al. 2014).

In EBHDs, type-I fibre mitochondrial content was reported to be ~ 35% higher compared with type-II fibres, whereas in NDs this did not differ between fibres (Elia et al. 2019b). Although there were no between-group differences in mitochondrial content, qualitative assessment of the relative myoglobin and mitochondrial protein distribution in muscle fibres revealed a stronger fluorescence intensity. Furthermore, there was a homogenous distribution of fluorescence intensity at the sub-sarcolemmal regions of type-I muscle fibres in the EBHDs compared with NDs. Such a homogenous distribution may potentially reduce the intracellular diffusion distance between oxymyoglobin and mitochondria. This novel observation, in combination with the higher myoglobin concentrations reported in the EBHDs type-I fibres, may suggest a greater reserve of readily available oxygen to support a similar (i.e., when compared with the type-I fibre mitochondrial content of NDs) mitochondrial respiration.

## Apnoea and the diving-response

During apnoea, the body undergoes a series of physiological modifications, known collectively as the diving-response, which adapts the body to the state of hypoxaemia and economically manages oxygen stores until respiration is re-established (Ferrigno et al. 1997; Foster and Sheel 2005; Gooden 1994; Lindholm and Lundgren 2009). The diving-response is primarily characterised by an initial parasympathetically-induced bradycardic response, which slows the depletion of oxygen stores (Asmussen and Kristiansson 1968; Hoiland et al. 2017; Landsberg 1975; Lin 1982) (Fig. 1). A selective sympathetically-induced peripheral vasoconstriction is also triggered in the arterioles (i.e., the peripheral and visceral capillary beds) of non-vital organs and the body’s extremities, with preferential redistribution of oxygenated blood towards the vital organs (Baković et al. 2003; Ferretti 2001; Kyhl et al. 2016; Mijacika and Dujic 2016) (Fig. 1). This results in a shift from predominantly aerobic to anaerobic metabolism in the non-essential organs, further lowering oxygen utilisation and compounding the diving-response-induced oxygen-conserving effect (Andersson et al. 2004, 2002). Taken together, these findings suggest that the diving-response plays a key role in reducing the severity of hypoxaemia across a given duration of apnoeic activity, by inducing a series of physiological modifications which economically manage oxygen utilisation.

The diving-response is evident in all humans; however, the magnitude of the predefined physiological components differs substantially across diving and non-diving populations. More experienced BHDs exhibit an earlier (10–20 s) and more pronounced bradycardic response (less than ~ 35 bpm) compared with NDs (50–70 s; ~ 50 bpm) (Elia et al. 2021b; Ferretti 2001; Ferretti et al. 1991; Lemaître et al. 2008; Schagatay et al. 2000). Similarly, during static apnoeas, EBHDs are known to exhibit greater increases in cerebral blood flow, higher cerebrovascular reactivity (Joulia et al. 2009; Vestergaard and Larsson 2019) and are able to withstand greater hypoxaemic and hypercapnic levels than NDs (Bain et al. 2016, 2018; Willie et al. 2015). Additionally, there is also evidence to suggest that trained BHDs exhibit a blunted ventilatory chemosensitivity to hypercapnia at rest and post-exercise (Delapille et al. 2001; Grassi et al. 1994; Roecker et al. 2014) that is distinct from scuba divers and controls (Roecker et al. 2014), in addition to displaying a reduced cerebral autoregulation compared with ND controls (Moir et al. 2019). Yet the nature of the latter adaptation remains to be determined whether it serves a protective purpose (i.e., preserving cerebral oxygen perfusion/delivery) or rather represents a more menacing phenomenon (i.e., exposing BHDs to a greater risk of cerebral hypoperfusion).

Although these disparities may be partly attributed to a training-induced effect (Joulia et al. 2002, 2003; Schagatay et al. 2000), accumulating evidence suggests they may also stem from selective genetic variations (Baranova et al. 2017; Ilardo et al. 2018). Baranova et al. (2017) observed that during a maximal apnoeic attempt, individuals with a combination of the C/C allele of the bradykinin receptor B2, D/D allele of the angiotensin-converting enzyme, and G/G polymorphism in the renin genes exhibited: (i) a more pronounced vasoconstriction, (ii) a lower blood supply to peripheral vessels, (iii) a lower pulse wave amplitude and (iv) lower pulse transit time values compared with heterozygous individuals. Conjointly, these genetic polymorphisms augment the release of angiotensin-II and suppress endothelial nitric oxide synthase through the degradation of bradykinin. Consequently, alterations in these vasoactive substances potentiate the noradrenergic diving-response-induced vasoconstriction and delay the recovery of vascular tone after the stimuli abates.

Therefore, the exaggerated diving-response observed in BHDs may result from training-induced stimuli and/or a natural selection of genetic polymorphisms that collectively protect them from intense hypoxaemia.

### Apnoea and face immersion

Apnoea, as the lone stimuli, is sufficient to elicit bradycardia, however, when apnoea is coupled with face immersion, a stronger bradycardial response is noticeable (Andersson et al. 2000; Ferrigno et al. 1997; Hayashi et al. 1997; Shamsuzzaman et al. 2014). The profound influences of facial cooling largely stem from stimulation of the trigeminal nerve activity (i.e., facial cold receptors innervated by the ophthalmic nerve), which evidently evoke a ‘trigeminocardiac reflex’, also referred to as the diving-reflex (Lemaitre et al. 2015). The magnitude of this reflex is highly variable and primarily depends on the water temperature the facial cold receptors are exposed to (Ferrigno et al. 1997; Schagatay and Holm 1996). Similarly, apnoea with face immersion has been evidenced to synergistically incite a stronger reduction in limb blood flow (i.e., finger and forearm) and greater muscle sympathetic nerve activity than individual stimuli for either apnoea or facial cooling (Heistad et al. 1968; Shamsuzzaman et al. 2014). Collectively, these data suggest that a stronger bradycardial, peripheral vasoconstriction and reduction in limb blood flow is evident when apnoeas are coupled with the stimulation of the facial cold receptors.

## Acute apnoea-induced humoral and stress-related responses

### Erythropoietin

Under hypoxic/hypoxaemic conditions, hypoxia-inducible factors stimulate the transcriptional activity of the erythropoietin (EPO) gene (Ebert and Bunn 1999; Haase 2010, 2013), subsequently actuating the synthesis of the glycoprotein hormone EPO by the kidneys and, to a lesser extent, the liver and brain—with the production of EPO being directly proportional to the level of systemic hypoxaemia (Eckardt et al. 1989; Elia et al. 2019a; Ge et al. 2002; Jelkmann 2011; Knaupp et al. 1992). EPO serves a pivotal role in maintaining oxygen homeostasis through inciting the process of erythropoiesis and consequently elevating RBC and haemoglobin mass (Haase 2010, 2013; Jelkmann 2011; Lundby et al. 2007; Rodríguez et al. 2000). While, to date, no study has evaluated the effect of voluntary apnoea on hypoxia-inducible factors expression, a number of studies have demonstrated that the systemic hypoxaemia brought about by apnoeic activity stimulates an acute increase in serum EPO (de Bruijn et al. 2008; Elia et al. 2019a, 2021b; Kjeld et al. 2015).

de Bruijn et al. (2008) first demonstrated that a series of fifteen dry static apnoeic repetitions were effective in increasing serum EPO concentrations (+ 1.38 mlU/L [+ 16%]) in NDs, 3 h after the last apnoeic bout, with values restored to baseline 5 h post. Similarly, Kjeld et al. (2015) reported significant increases in EPO (+ 1.8 mIU/L) 3 h following a single bout of a combined maximal static and dynamic apnoeic attempt in a group of EBHDs. More recently, the first distinction between the individual erythropoietic effects of repeated static and dynamic apnoeas was provided by Elia et al. (2019a). In EBHDs, NDs and controls, a series of ten repeated maximal static apnoeic bouts with whole-body immersion did not significantly affect serum EPO concentrations in relation to baseline (Elia et al. 2019a). However, in elite exponents only, concentrations were elevated at 30 min (+ 60%; + 3.97 mIU/L) and 180 min (+ 63%; + 4.02 mIU/L) following just five maximal dynamic apnoeic bouts. This increase was, however, not mirrored in ND, potentially due to the lower degree of hypoxaemia experienced by the NDs than the EBHDs. The strong association between end-apnoeic peripheral oxygen saturation levels and peak post-apnoeic serum EPO (*r* = − 0.49) seems to support this notion.

Considering the key role that EPO serves in the process of erythropoiesis (Eckardt et al. 1989; Jelkmann 2011), a number of studies have explored the possibility of apnoea-induced hypoxaemia in enhancing erythrocyte concentrations. Engan et al. (2013) first signified that 2-weeks of daily dry static apnoeic training (i.e., ten maximal apnoeic bouts, in total) was effective in increasing resting reticulocyte concentrations. These findings highlighted the possible utility of apnoeic training as a sufficient stimulus for erythropoiesis. However, conflicting evidence has since been published regarding the effect of apnoeic training on haematology (Bouten et al. 2019; Elia et al. 2021a; Fernandez et al. 2019); with Bouten et al. (2019) reporting significant increases (+ 6 g/L) in haemoglobin concentrations following 8-weeks of daily dry static apnoeic training (i.e., five maximal apnoeic bouts per day) whereas both Fernandez et al. (2019) and Elia et al. (2021a) failed to record any gains in haemoglobin and RBC following 22-weeks (i.e., structured apnoea program) and 6-weeks (i.e., ten maximal dynamic apnoeic bouts, four times per week) of apnoeic training, respectively. However, it is worth noting that Bouten et al. (2019) did not assess total haemoglobin mass nor plasma volume. Taking into consideration that haemoglobin concentrations are prone to plasma volume changes (Otto et al. 2013, 2017), it is unclear whether the increases reported by Bouten et al. (2019) following apnoeic training reflect a true improvement in haemoglobin concentrations. Therefore, it remains to be elucidated whether apnoea-induced increases in serum EPO concentrations are sufficient to stimulate erythropoiesis and ultimately translate to chronic increases in RBC and haemoglobin mass.

### Oxidative stress and antioxidant enzyme activity

Once breathing is reinstated following a hypoxaemic bout, reactive hyperaemia and systemic reoxygenation results in increased free radical and associated reactive oxygen species (ROS) production alongside a state of oxidative stress (Li and Jackson 2002). Similarly, repeated maximal apnoeic attempts have been documented to upregulate the production of ROS in the systemic circulation (Joulia et al. 2002, 2003), accentuating that repeated apnoeic bouts can aggravate systemic oxidative stress levels (Mrakic-Sposta et al. 2019; Sureda et al. 2004b, 2015; Theunissen et al. 2013). Interestingly, following sustained static and dynamic apnoeic activity EBHDs exhibited lower post-exercise blood acidosis and oxidative stress (i.e., thiobarbituric acid reactive substances [TBARS], reduced glutathione [GSH] and reduced ascorbic acid) compared with NDs, despite attaining significantly greater (302 ± 30 s vs*.* 104 ± 10 s) apnoeic performances (Joulia et al. 2002)—an observation that was later ascribed to a training-induced adaptation (Joulia et al. 2003).

The lower post-apnoeic oxidative stress documented in EBHDs may, at least in part, stem from an enhanced antioxidant enzyme activity and resistance to antioxidant depletion (Bulmer et al. 2008; Sureda et al. 2004a, b). Indeed, 1 h following a series of repeated apnoeic dives (~ 55 dives) erythrocyte catalase and lymphocyte superoxide dismutase (SOD) were enhanced; a response which likely protected erythrocytes against any oxidative damage (Sureda et al. 2006). Similarly, following a single, submaximal dynamic apnoeic attempt, Rousseau et al. (2006) reported significant increases in plasma glutathione peroxidase (GPx-3) activity and blood GSH, whereas no changes were observed in erythrocyte SOD, erythrocyte glutathione peroxidase (GPx), blood oxidised glutathione and TBARS concentrations. Moreover, after five static apnoeic repetitions, circulating antioxidant concentrations (i.e., bilirubin and uric acid) were reduced while antioxidant enzyme activity (i.e., SOD and GPx) was enhanced without causing an increase in oxidative stress (i.e., malondialdehyde) (Bulmer et al. 2008). More recently, Sureda et al. (2015) evaluated for the first time the cumulative effects of daily apnoeic exposures on ROS generation, oxidative stress and antioxidant enzyme activity. Following a series of repeated apnoeic dives (i.e., ~ 200 dives performed over a period of 5 days) xanthine oxidase activity, an enzyme responsible for the generation of ROS, was significantly elevated (+ 98%) (Sureda et al. 2015). However, concomitant increases in antioxidant enzyme activity (i.e., catalase, GPx, glutathione reductase and SOD) and protein levels (i.e., catalase), primarily observed 15 h after the last hypoxaemic bout, inhibited xanthine oxidase-derived ROS generation (i.e., supported by the lack of changes in malondialdehyde and protein carbonyl derivates levels) and protected cells from oxidative damage. Collectively, these findings illustrate that acute apnoea enhances oxidative stress in contrast to repeated apnoeas that appear to demonstrate an attenuation of oxidative stress. This may represent a protective physiological adaptation to the endogenous antioxidant defence system, capable of opposing excessive ROS and maintaining redox balance.

### Neuronal stress

Emerging evidence indicates that maximal apnoeic bouts are associated with transient disruption of the blood–brain barrier (Andersson et al. 2009; Bain et al. 2018; Matsuo et al. 2014) and neuronal-parenchymal damage (Gren et al. 2016). In nine BHDs, Andersson et al. (2009) reported significant increases (~ 26%) in serum S100β at the end of a maximal static apnoea, with values being restored to baseline ~ 2 h post; indicative of a potential perturbation of the blood–brain barrier. A subsequent study by Kjeld et al. (2015) revealed significant increases (~ 70%) in neuron-specific enolase (NSE), but failed to record any increases in S100β levels 3 h following a combined bout of static and dynamic apnoea, possibly owing to the short half-life of S100β (~ 2 h) (Thelin et al. 2017). Separately, in competitive BHDs, static apnoea increased plasma concentrations of total tau and amyloid β42, whereas no changes were observed in neurofilament light protein or S100β (Gren et al. 2016). More recently, immediately after a maximal apnoeic bout S100β concentrations were significantly elevated (~ 40%), while NSE and human myelin basic protein (a specific biomarker for cerebral axonal damage) did not change (Bain et al. 2018). These findings were interpreted as a minor disturbance of the blood–brain barrier, but not enough to cause neuronal-parenchymal damage. Presently, it is unclear whether these transient increases are simply reflective of functional physiological responses, or rather depict a maladaptive phenomenon. Moreover, to the best of our knowledge, no study has examined the neurological strain imposed by a series of repeated maximal apnoeic bouts (i.e., three or more repetitions). Considering that intermittent hypoxaemia (i.e., hypoxia and reoxygenation) is evinced to be capable of disrupting the permeability of the blood–brain barrier as well as interfering with the critical intercellular tight junction protein complexes (Almutairi et al. 2016; Kaur and Ling 2008; Lochhead et al. 2010), physiological modifications that could, in the long-term, lead to neuronal dysfunction and degeneration, together reinforce the necessity for further studies to be undertaken.

### Cardiac stress

Following a static apnoeic attempt and a 5 h spearfishing competition, copeptin and ischemia-modified albumin (IMA)—all representative markers of acute and hypoxaemic stress—were elevated above baseline (Joulia et al. 2015; Marlinge et al. 2019). Interestingly, the recorded IMA increases were greater than those denoted in chronic cardiac failure patients (Franceschi et al. 2009) and during acute coronary ischemia (Bali et al. 2008; Lee et al. 2007); thus highlighting the severity of the physiological stress encountered by BHDs during apnoea. More importantly, following repeated apnoeic dives the cardiac injury markers cardiac troponin I and brain natriuretic peptide were significantly elevated (+ 275% and + 229%, respectively) from basal levels; a response that was not evident following a single static apnoeic attempt, nor after a combined bout of static and dynamic apnoeas (Kjeld et al. 2015; Marlinge et al. 2019).

Considering the substantial physiological stress experienced by BHDs during voluntary apnoeic efforts, the question then arises as to whether chronic exposures to repeated, transient apnoeic interventions could lead to health implications. Accordingly, the following section will seek to delineate the possible maladaptation(s) of chronic apnoeic training.

## Maladaptation(s) associated with chronic apnoeic training

### Neurocognition

There is compelling evidence highlighting the deleterious effects of acute and chronic exposure to hypoxia across a range of cognitive and behavioural performances (Caine and Watson 2000; Hornbein et al. 1989; Truszczyński et al. 2009). As previously discussed, even transient apnoea-hypoxaemia gives rise to increased serum S100β (a marker of cerebral ischemia and brain damage) (Andersson et al. 2009; Bain et al. 2018), plasma NSE (a marker of acute neuronal damage) (Kjeld et al. 2015) and alters amyloid metabolism by increasing plasma total tau and amyloid β42 (i.e., reflecting neuronal damage/dysfunction) (Gren et al. 2016). One may, therefore, argue that repeated exposures to such transient, severe hypoxaemia could lead to chronic negative consequences in BHD populations (Fig. 2).

In five EBHDs, single photon emission computed tomographic scans revealed brain abnormalities, demonstrating both large focal and/or diffuse areas of hypo- and hyper-perfusion in the frontal and temporal lobes and cerebellar hemispheres (Potkin and Uzsler 2006). Moreover, brain magnetic resonance imaging (MRI) scans in 11 out of 12 male Ama divers revealed cerebral ischemic lesions which were predominately situated in the cortex and subcortical white matter—characteristics that are indicative of circulatory disturbance at the corticomedullary junctional area of cerebral arteries (Kohshi et al. 2014) (Fig. 2). These MRI findings are so-called low-flow cerebral infarctions resulting from low perfusion pressure in terminal supply areas which the authors argued were not the result of aging. Contrastingly, in 17 EBHDs, Doerner et al. (2018) did not observe any cerebral (i.e., acute or sub-acute cerebral ischemia) morphological alterations at baseline nor at follow-up after 1 year compared both intra-individually and with healthy controls. Taken together, the inconsistent findings across these studies highlight the necessity for more research to be undertaken in larger cohorts to further our understanding of the chronic effects of breath-hold diving on cerebral integrity.

Contrary to the common assumption that breath-hold diving holds no risk for decompression sickness, it has been shown that multiple repeated breath-hold dives can produce venous gas emboli (Cialoni et al. 2016; Lemaître et al. 2014; Spencer and Okino 1972). Symptoms in BHDs consistent with decompression sickness have also been reported both after several repeated dives and following very deep single breath-hold dives [see Lemaitre et al. (2009) for review]. Decompression sickness caused by breath-hold dives tends to affect the central nervous system. The reason for this is unknown, although arterialisation of venous gas emboli, either via pulmonary shunts (Schipke and Tetzlaff 2016), or due to compression and release of venous gas emboli lodged in the pulmonary capillaries during repeated dives are possible mechanisms. Irrespective of the mechanisms, it is clear that when investigating cerebral pathology in EBHD the effects of asymptomatic or symptomatic embolic events must be differentiated from the effects of repeated hypoxia. Therefore, the prior activities of EBHD must be carefully explored and reported. It would be especially interesting to draw comparisons between EBHD who only carry out static apnoeas and/or dynamic breath-hold dives but not deep dives, and possibly distinguish them from EBHD who perform both repeated and deep dives.

To date, conflicting evidence exists concerning the long-term sequalae of apnoeic training on neurocognitive health. Ridgway and McFarland (2006) failed to report any neurocognitive impairments in 21 BHDs compared with non-diving controls using a series of neuropsychological tests. Similarly, apnoea-induced hypoxaemia and hypercapnia did not seem to impair neurocognitive nor visual/cognitive processing stages (Ratmanova et al. 2016; Steinberg and Doppelmayr 2019). On the other hand, during a Stroop test, EBHD took longer to complete an interference card, made more errors, and had a lower total interference score than novice BHDs and controls (Billaut et al. 2018). More importantly, the time taken to complete the card was positively associated with maximal static apnoea duration (*r* = 0.73) and the number of years engaged in breath-hold diving training (*r* = 0.79). These findings signify that long-term participation in breath-hold diving, may give rise to short-term memory impairments. The discrepancies among the findings of these studies may relate to the training background and apnoeic characteristics of the BHDs recruited. However, the growing popularity of the sport necessitates that further longitudinal studies are conducted to elucidate the potential chronic maladaptations to apnoeic-related activities.

### Cardiovascular and arterial modifications

During prolonged apnoeas, peripheral vasoconstriction from elevated sympathetic nerve activity, in addition to the onset of involuntary diaphragmatic contractions (Fagoni et al. 2017), progressively lead to increases in mean arterial pressure (~ 35–55%) (Breskovic et al. 2011; Perini et al. 2008; Willie et al. 2015). Moreover, during dry maximal static apnoeas, Doerner et al. (2018) recorded dilations of the left ventricle as well as reductions in the left ventricular ejection fraction accompanied by an increased left ventricular systolic volume—a response that was evident in EBHDs but not in NDs. Considering that both persistent hypertension and cardiac dilation can lead to cardiac remodelling with fibrotic changes (Assomull et al. 2006), one wonders whether chronic apnoeic training may give rise to an increased risk of cardiovascular disease.

Resting echocardiograms and 24 h continuous electrocardiogram monitoring did not unveil any cardiac abnormalities in 16 competitive BHDs (Zelenkova and Chomahidze 2016). Similarly, using magnetic resonance imaging, neither late gadolinium enhancement imaging nor T1-mapping and its derived parameters showed significant cardiac morphological alterations (i.e., myocardial scars or fibrotic changes) at baseline, nor at follow-up after 1 year compared intra-individually and with healthy controls (Doerner et al. 2018). Moreover, a cross-sectional study that investigated the arterial stiffness of 115 female lifelong Ama BHDs (i.e., 38 ± 8 years practicing apnoea) with age-matched physically active adults demonstrating similar arterial stiffness, cardio-ankle vascular index and β-stiffness index (Tanaka et al. 2016). Therefore, in totality, despite some findings that indicate acute cardiac disturbances (Joulia et al. 2015; Marlinge et al. 2019), the current evidence suggests that long-term participation in apnoea-related activities does not affect cardiac health nor vascular integrity (Fig. 2).

### Bone tissue

Mechanical loading has a vital impact on bone remodelling and bone mineral density (BMD) (Chahal et al. 2014; Lanyon and Rubin 1984), with bone responding adaptively to its habitual loading environment, for example the contractile forces applied by skeletal muscle (Hart et al. 2017). Although the high-pressure conditions associated with diving may be osteogenic, this stimulus appears to be mitigated by the weight-supported underwater environment (Hwang et al. 2006). Male EBHDs were shown to possess significantly lower bone mineral content (BMC) than age- and morphometry-matched controls (3.1 ± 0.2 kg vs. 3.7 ± 0.4 kg) (Kjeld et al. 2018) (Fig. 2). This was also the case for BMD (1.3 ± 0.1 kg/m^2^ vs. 1.4 ± 0.1 kg/m^2^) and *T*/*Z*-score (0.6 ± 0.8 vs. 2.3 ± 0.8). In support, Fernandez et al. (2017) reported comparable BMD values of 1.28 ± 0.09 g/cm^2^ in BHDs (Fig. 2). However, it is important to note that comparisons of dual-energy x-ray absorptiometry (DXA) measurements from different machines should be made with a degree of caution (Gillette-Guyonnet et al. 2003; Saarelainen et al. 2016). Furthermore, both of these studies measured total-body BMC and BMD, whereas the use of additional measurement sites, such as the proximal femur, may provide more information with relation to the risk of osteoporotic fracture (Lu et al. 2001). Lumbar spine and femoral neck BMD and *T*-scores were similar in female Ama BHDs compared to NDs, yet these participants were postmenopausal and older (~ 70 years) than those in comparable literature (Seo et al. 2018). Importantly, in both groups, the reported *T*-scores were suggestive of osteopenia (Karaguzel and Holick 2010) and approached the threshold for osteoporosis in this population [≤ −2.5; Kanis (1994), Park et al. (2016)], thus indicating that substantial cumulative exposure to the diving environment does not confer benefits with regards to BMD at the lumbar spine and proximal femur. In a younger (~ 54 year) population of female divers, with ~ 34 years of diving experience, greater BMD values were seen for the total hip and femoral neck areas compared to controls (Hwang et al. 2006). Whilst the femoral neck *T*-score  was in the healthy range in premenopausal divers, postmenopausal divers and NDs demonstrated *T*-scores indicative of osteopenia. Noticeably, age was a predictor of proximal femur BMD in BHDs and controls, revealing a more rapid decrease in BMD in divers. Furthermore, BMD was reduced in proportion to time spent in the water (Hwang et al. 2006).

Prolonged and repeated exposure to hypercapnic conditions may significantly impact bone metabolism. For example, untreated chronic obstructive pulmonary disease patients with hypercapnia exhibited lower BMD than eucapnic comparators (Dimai et al. 2001), a difference that was partially attributed to increased bone resorption (i.e., the breakdown of bone tissue by osteoclasts to liberate calcium). The decreased pH of the blood brought about by hypercapnia may also drive this process, whilst inhibiting mineral deposition by osteoblasts (i.e., bone formation) in a seemingly reciprocal manner (Arnett 2010). Interestingly, the resorptive activity of osteoclasts is not impaired in hypoxia, however, the growth and differentiation of osteoblasts is inhibited (Arnett 2010). Therefore, the combined effects of hypoxaemic and hypercapnic exposure, as well as the weight-supported environment in competitive and habitual BHDs may influence bone metabolism to varying degrees, thus partially explaining the differences reported above. In light of this evidence, the use of prophylactic measures for the prevention of osteoporosis [i.e., calcium and vitamin D supplementation, weight-bearing exercise and/or antiresorptive agents, (Kling et al. 2014)] may be justified in BHDs, particularly for older individuals with a more substantial history of diving activity. However, the nature and efficacy of such interventions in diving populations is yet to be explored.

Taken together, the current evidence concerning the impact of prolonged breath-hold diving on BMC and BMD is equivocal, with conflicting findings depending on the measurement site and/or population in question. Therefore, these aspects of bone health should be explored further in longitudinal investigations of elite and occupational diving populations.

### Renal health

To our knowledge, the only study to have explored the impact of long-term breath-hold diving on kidney function was in female habitual Korean divers, using estimates of glomerular filtration rate (Oh et al. 2017). After matching for propensity scores (*n* = 715 per group), chronic kidney disease prevalence was significantly higher in BHDs compared with non-diving controls (12.6% vs*.* 8.0%). Multivariate analyses revealed significant associations between diving activity and chronic kidney disease risk in an unmatched cohort. In the propensity score-matched cohort, diving remained the independent risk factor for chronic kidney disease, even following adjustment for multiple covariates. Moreover, following a single, sled-assisted dive to 40 m, creatinine concentrations increased (+ 40%), suggesting a possible early renal dysfunction (Mrakic-Sposta et al. 2019). Conjointly, these findings provide evidence that sustained, long-term breath-hold diving activity may lead to a deterioration in kidney function (Fig. 2). The causality of this association needs to be explored further in longitudinal studies of BHDs.

## Conclusions

This review has provided (*i*) an overview of the pertinent physiological traits and mechanisms that govern an individual’s apnoeic capabilities, (*ii*) outlined the humoral and stress responses to maximal apnoeic bouts and (*iii*) delineated the physiological (mal)adaptations to chronic apnoeic training. The present evidence suggests that BHDs demonstrate a more pronounced diving-response than NDs while elite exponents also display beneficial adaptations in blood and skeletal muscle, primarily as a consequence of exposure to training-induced stimuli. Moreover, BHDs possess an ability to endure distinct acute cerebrovascular and neuronal stressors, the physiological basis of which (i.e., training-induced or as a consequence of genetic disposition) is less understood. Over the long-term, sustained breath-hold diving activity may pose ramifications for renal health, whereas the impact on bone tissue, neurocognition and cardiovascular function is less clear.

### Future directions

This review highlights the necessity for further cross-sectional and longitudinal studies to be performed, with the goal of advancing our fundamental understanding of the maladaptation(s) associated with chronic apnoeic stress as well as the influence of training-induced and genetic factors on apnoeic performance. Preliminary evidence has posed considerations for neurocognition, renal and bone health in BHDs. Considering the growing popularity of the sport and the increasing number of people pursuing breath-hold diving as a competitive and/or recreational activity, enhanced understanding of the (mal)adaptation(s) of chronic apnoeic training is paramount from a safety and medical standpoint.

## Abbreviations

BHD: Breath-hold diver
BMC: Bone mineral content
BMD: Bone mineral density
CSA: Cross-sectional area
DXA: Dual-energy x-ray absorptiometry
EBHD: Elite breath-hold diver
EPO: Erythropoietin
GPx: Erythrocyte glutathione peroxidase
GPx-3: Plasma glutathione peroxidase
GSH: Reduced glutathione
IMA: Ischemia-modified albumin
Mb: Myoglobin
MCV: Mean cell volume
MRI: Magnetic resonance imaging
ND: Non-diver
NSE: Neuron-specific enolase
PaCO_2_: Arterial carbon dioxide partial pressure
PaO_2_: Arterial oxygen partial pressure
R_95_: Diffusion distance
RBC: Red blood cell
ROS: Reactive oxygen species
SOD: Superoxide dismutase
TBARS: Thiobarbituric acid reactive substances
TLC: Total lung capacity

## Footnotes

1 After a full inhalation to TLC, a mouthful of air with the glottis closed is compressed by the oropharyngeal muscles and then forced into the lungs, opening the glottis just for the gulping manoeuvre. This glossopharyngeal insufflation manoeuvre is repeated on several occasions until a sensation of fullness occurs.
